# Changes in Surface-Charge Density of Blood Cells After Sudden Unexpected Death

**DOI:** 10.1007/s00232-012-9428-4

**Published:** 2012-04-20

**Authors:** Joanna Kotyńska, Aneta D. Petelska, Michał Szeremeta, Anna Niemcunowicz-Janica, Zbigniew A. Figaszewski

**Affiliations:** 1 Institute of Chemistry, University of Bialystok, Al. J. Pilsudskiego 11/4, 15-443 Bialystok, Poland; 2 Department of Forensic Medicine, Medical University of Bialystok, Waszyngtona St. 13, 15-230 Bialystok, Poland; 3 Laboratory of Electrochemical Power Sources, Faculty of Chemistry, University of Warsaw, Pasteur St. 1, 02-093 Warsaw, Poland

**Keywords:** Erythrocytes, Microelectrophoresis, pH measurement, Sudden unexpected death, Surface charge density, Thrombocytes

## Abstract

The objective of the investigation was evaluation of postmortem changes of electric charge of human erythrocyte and thrombocyte membranes after sudden unexpected death. The surface charge density values were determined on the basis of the electrophoretic mobility measurements of the cells carried out at various pHs of electrolyte solution. The interactions between both erythrocyte and thrombocyte membranes and electrolyte ions were studied. Values of parameters characterizing the membrane—that is, the total surface concentrations of both acidic and basic groups and their association constants with solution ions—were calculated on the basis of a four-equilibria mathematical model. The model was validated by comparison of these values to experimental data. We established that examined electric properties of the cell membranes are affected by sudden unexpected death. Postmortem processes occurring in the cell membranes can lead to disorders of existing equilibria, which in turn result in changes in values of all the above-mentioned parameters.

## Introduction

The surface electric charge density of biological membranes is an important parameter for the maintenance of normal physiological functions of cells. It controls several processes in biological membranes, such as membrane-bound enzymes, insertion of newly synthesized proteins into membranes, and host–pathogen interactions (Yermiyahu et al. 1999). Apart from this, knowledge of surface electric charge values can provide valuable information about the equilibria existing within the membrane and between the membrane and its surroundings.

Biological membranes are negatively charged in physiological pH, mainly as a result of the presence of acidic phospholipids; about 10–20 % of total membrane lipids are anionic ones. Other membranes constituents such as proteins or gangliosides also contribute to the negative charge (Nałęcz and Wojtczak 1982; Gennis 1989). Because the membrane is exposed to surrounding aqueous buffer, specific interactions with outer medium components occur. The resulting equilibria, in which charged groups of membrane components and solutions ions are involved, can be affected by different factors and processes leading to a membrane surface charge density variation. The parameter is also influenced by membrane composition, ionic strength of electrolytes, and solution pH (Deshiikan and Papadopoulos 1998). Changes in pH alter the surface charge of a membrane, with the membrane becoming more positive at a lower pH and more negative at a higher pH (Mullet et al. 1997; Dobrzyńska et al. 2006; Petelska et al. 2012). In vitro, the surface charge density of biological membranes can be changed either by adding, for example, ionizable surfactants (McLaughlin and Harary 1976) or divalent ions (Mg^2+^, Ca^2+^) (Barber 1980) to the membrane suspension, or by altering the membrane lipid composition (Nałęcz et al. 1980). In certain conditions, the surface charge density may be subject to in vivo modification. It was observed that changes in cell surface components (e.g., proteoglycans or sialic acids, which are typical for cancer transformations or other pathologies) are accompanied by changes in surface charge of a cell membrane (Dobrzyńska et al. 2010; Monteggia et al. 2000). Therefore, it seems that variation in membrane surface charge density can commonly occur in living cells, and the resultant membrane charge is the result of a number of various overlapping processes (Nałęcz and Wojtczak 1982).

The most common cause of sudden unexpected death is cardiovascular disease due to sudden cardiac death, acute myocardial ischemia resulting from coronary atherosclerosis, or lethal arrhythmia (Langlois 2009). Biochemical processes, which are controlled in the living organism, can be significantly altered during the course of disease (Kała and Chudzikiewicz 2003). Concentrations of many substances that occur in living organism at normal levels can be influenced after sudden unexpected death. After cessation of circulation, the physiology of the blood and the vascular system completely changes. In cadaveric blood, a rapid increase of catecholamine levels is also observed, with a higher level of adrenaline than noradrenalin (Takeichi et al. 1984), a high level of K^+^ ions (Takeichi et al. 1985), and a lower level of pH, and base excess (BE) and HCO_3_^−^ ions (Takeichi et al. 1986). In the early postmortem phase, the dead body is exposed to autolysis—that is, dissolution of organs under the impact of endogenous enzymes. The autolysis of blood (hemolysis) can generate many new chemical compounds, leading to disorders of equilibria describing the specific membranes. Quantitative estimates of the equilibria and numerical determination of the membrane characterizing parameters are extremely important for the interpretation of changes in physicochemical processes. Appearance of new groups on the surface of membranes or loss of existing ones causes changes not only the membrane’s electric charge, but also the total surface concentrations of both acidic and basic groups and their association constants with solution ions.

This work continues the systematic study of electrical properties of membranes both model and biological realized by Figaszewski and coworkers (Dobrzyńska et al. 2007, 2008; Kotyńska et al. 2008; Naumowicz et al. 2006; Petelska and Figaszewski 2011; Szachowicz-Petelska et al. 2010). We examined postmortem changes of the surface charge of erythrocyte and thrombocyte membranes after sudden unexpected death. Our experiment was performed using the microelectrophoresis method, which is one of the basic analytical tools for biological studies. The electrophoretic mobility measurements were done over the pH range 2–11. On the basis of a mathematical model describing the equilibria between a cell membrane and surrounding ions, the parameters characterizing the equilibria were determined. In our opinion, the quantitative description of cell membrane surface properties can help in interpreting and understanding the processes that take place on biological membrane surfaces after sudden unexpected death.

## Theory

The model, which has been presented in full detailed by Dobrzyńska et al. (2006), assumes that dependence of surface charge density of the cell membrane on the pH of electrolyte solution can be described with the help of four equilibria. Two are connected with positive groups (e.g., phospholipids or proteins and sodium and hydrogen ions), and two concern the negative species of phospholipids or proteins and hydroxide and chloride ions. The H^+^, OH^−^, Na^+^, and Cl^−^ ions are adsorbed at the cell membrane (erythrocyte or thrombocyte), and the adsorption equilibria (Eqs. 1–4) can be presented in the following form:

1 $$ {\text{A}}^{ - } + {\text{ H}}^{ + } \Leftrightarrow {\text{AH}} $$

2 $$ {\text{A}}^{ - } + {\text{ Na}}^{ + } \Leftrightarrow {\text{ANa}} $$

3 $$ {\text{B}}^{ + } + {\text{ OH}}^{ - } \Leftrightarrow {\text{BOH}} $$

4 $$ {\text{B}}^{ + } + {\text{ Cl}}^{ - } \Leftrightarrow {\text{BCl}} $$

Therefore, the association constants of the H^+^, Na^+^, OH^−^, and Cl^−^ ions with functional groups are expressed in the following manner (Dobrzyńska et al. 2006, 2007):

5 $$ K_{\text{AH}} = \frac{{a_{\text{AH}} }}{{a_{{{\text{A}}^{ - } }} \cdot a_{{{\text{H}}^{ + } }} }} $$

6 $$ K_{\text{ANa}} = \frac{{a_{\text{ANa}} }}{{a_{{{\text{A}}^{ - } }} \cdot a_{{{\text{Na}}^{ + } }} }} $$

7 $$ K_{\text{BOH}} = \frac{{a_{\text{BOH}} }}{{a_{{{\text{B}}^{ + } }} \cdot a_{{{\text{OH}}^{ - } }} }} $$

8 $$ K_{\text{BCl}} = \frac{{a_{\text{BCl}} }}{{a_{{{\text{B}}^{ + } }} \cdot a_{{{\text{Cl}}^{ - } }} }} $$

where, $K_{\text{AH}} ,\,K_{\text{ANa}} ,\,K_{\text{BOH}}$ and $K_{\text{BCl}}$ are association constants; $a_{\text{AH}}$, $a_{\text{ANa}}$, $a_{{{\text{A}}^{ - } }}$, $a_{\text{BOH}}$, $a_{\text{BCl}}$, and $a_{{{\text{B}}^{ + } }}$ are surface concentrations of corresponding groups on the membrane surface; $a_{{{\text{H}}^{ + } }}$, $a_{{{\text{Na}}^{ + } }}$, $a_{{{\text{OH}}^{ - } }}$ and $a_{{{\text{Cl}}^{ - } }}$ are $a_{{{\text{Cl}}^{ + } }}$ are volume concentrations of solution ions.

The concentrations balances are expressed as follows (Dobrzyńska et al. 2006):

9 $$ C_{\text{A}} = a_{{{\text{A}}^{ - } }} + a_{\text{AH}} + a_{\text{ANa}} $$

10 $$ C_{\text{B}} = a_{{{\text{B}}^{ + } }} + a_{\text{BOH}} + a_{\text{BCl}} $$

where *C*_A_ is the total surface concentration of the membrane acidic groups and *C*_B_ is the total surface concentration of the membrane basic groups.

Surface charge density of the membrane is given by the following equation (Dobrzyńska et al. 2006):

11 $$ \delta = \left( {a_{{{\text{B}}^{ + } }} - a_{{{\text{A}}^{ - } }} } \right) \cdot F $$

where

$$ F = 96487\left[ {\frac{C}{\text{mol}}} \right] - {\text{Faraday constant}} $$

Elimination of, $a_{\text{AH}}$, $a_{\text{ANa}}$, $a_{{{\text{A}}^{ - } }}$, $a_{\text{BOH}}$, $a_{BCl}$_,_ and $a_{{B^{ + } }}$ from the above equations yields the following formula (Dobrzyńska et al. 2006):

12 $$ \frac{\delta }{F} = \frac{{C_{\text{B}} }}{{1 + K_{\text{BOH}} a_{{{\text{OH}}^{ - } }} + K_{\text{BCl}} a_{{{\text{Cl}}^{ - } }} }} - \frac{{C_{\text{A}} }}{{1 + K_{\text{AH}} a_{{{\text{H}}^{ + } }} + K_{\text{ANa}} a_{{{\text{Na}}^{ + } }} }} $$

Determination of the searching parameters requires a simplification of the above equation to a linear form at high H^+^ (*a*_H+_ → ∞) and low H^+^ (*a*_H+_ → 0) concentrations, which appeared previously (Dobrzyńska et al. 2006). In the former case, Eq. 12 was rewritten as a decreasing exponential function of H^+^ concentration (Eq. 13), and in the latter case, it was rewritten as an increasing exponential function of H^+^ concentration (Eq. 14) (Dobrzyńska et al. 2006).

13 $$ \frac{\delta }{F} = \frac{{C_{\text{B}} a_{{{\text{H}}^{ + } }} }}{{a_{{{\text{H}}^{ + } }} (1 + K_{\text{BCl}} a_{{{\text{Cl}}^{ - } }} ) + K_{\text{BOH}} K_{\text{W}} }} - \frac{{C_{\text{A}} }}{{K_{\text{AH}} a_{{{\text{H}}^{ + } }} + K_{\text{ANa}} a_{{{\text{Na}}^{ + } }} + 1}} $$

14 $$ \frac{\delta }{F} = \frac{{C_{\text{B}} a_{{{\text{H}}^{ + } }} }}{{K_{\text{BOH}} K_{\text{W}} + a_{{{\text{H}}^{ + } }} (1 + K_{\text{BCl}} a_{{{\text{Cl}}^{ - } }} )}} - \frac{{C_{\text{A}} }}{{K_{\text{ANa}} a_{{{\text{Na}}^{ + } }} + 1 + K_{\text{AH}} a_{{{\text{H}}^{ + } }} }} $$

The numerator of each term in Eq. 13 was divided by the denominator to yield two terms. These operations resulted in a linear equation in the *a*_H+_ and $\frac{{\delta a_{{{\text{H}}^{ + } }} }}{F}$ coordinate system, which was correct for high hydrogen ion concentrations (*a*_H+_ → ∞) (Dobrzyńska et al. 2006):

15 $$ \frac{{\delta a_{{{\text{H}}^{ + } }} }}{F} = \frac{{C_{\text{B}} }}{{1 + K_{\text{BCl}} a_{{{\text{Cl}}^{ - } }} }}a_{{{\text{H}}^{ + } }} - \left( {\frac{{C_{\text{B}} K_{\text{BOH}} K_{\text{W}} }}{{(1 + K_{\text{BCl}} a_{{{\text{Cl}}^{ - } }} )^{2} }} + \frac{{C_{\text{A}} }}{{K_{\text{AH}} }}} \right) $$

Applying the same procedure to Eq. 14 resulted in a linear equation in the $\frac{1}{{a_{{{\text{H}}^{ + } }} }}$ and $\frac{\delta }{{Fa_{{{\text{H}}^{ + } }} }}$ coordinate system, which was correct for low hydrogen ion concentrations (*a*_H+_ → 0) (Dobrzyńska et al. 2006):

16 $$ \frac{\delta }{{Fa_{{{\text{H}}^{ + } }} }} = - \left( {\frac{{C_{\text{A}} }}{{1 + K_{\text{ANa}} a_{{{\text{Na}}^{ + } }} }}} \right)\frac{1}{{a_{{{\text{H}}^{ + } }} }} + \left( {\frac{{C_{\text{B}} }}{{K_{\text{BOH}} K_{\text{W}} }} + \frac{{C_{\text{A}} K_{\text{AH}} }}{{(1 + K_{\text{ANa}} a_{{{\text{Na}}^{ + } }} )^{2} }}} \right) $$

The coefficients describing these linear functions may be easily obtained by linear regression and subsequently applied to calculate the parameters. The calculation of the parameters; $C_{\text{A}}$, $C_{\text{B}}$, $K_{\text{AH}}$, and $K_{BOH}$ is possible as a result of knowledge of the association constants-$K_{ANa}$, and $K_{\text{BCl}}$ obtained for phosphatidylcholine liposome membrane (Dobrzyńska et al. 2007). Defining the value of these parameters permits the calculation of the theoretical cell membrane surface charge from Eq. 12 for comparison to experimental data.

## Materials and Methods

### Materials

Approval for this study was granted by the Ethics Review Board of the Medical University of Bialystok (No. R-I-002/533/2010). Blood was obtained from all individuals during autopsies performed at the Forensic Medicine Department at the Medical University of Bialystok in the year 2010. The subject of the examination was based on 28 cases of selective sudden unexpected death (20 men, eight women; mean age 34.3 years; range 22–41 years; all causes of death due to sudden cardiac death, without coagulation disorders). Blood was routinely obtained from the femoral vein and placed into chemically and biologically clean glass containers, then donated to the Department of Electrochemistry at the University of Bialystok. The donated samples were comparatively analyzed to the control samples taken from live individuals from the blood service center in Bialystok.

#### Preparation of Erythrocytes from Blood

Erythrocytes were isolated from blood by centrifugation at 900×*g* for 8 min at room temperature. The supernatant thrombocyte-rich plasma was removed and saved for subsequent processing, while the erythrocytes were washed three times with isotonic saline (0.9 % NaCl) at 3,000×*g* for 15 min. After the final wash, the erythrocyte pellet was resuspended in isotonic saline for electrophoretic measurement.

#### Preparation of Thrombocytes from Plasma

The thrombocyte-rich plasma was centrifuged at 900×*g* for 8 min. The supernatant plasma was removed and discarded. The thrombocyte pellet was washed three times with isotonic saline by centrifugation at 3,000×*g* for 15 min. After the final wash, the thrombocytes were resuspended in isotonic saline for electrophoretic measurement.

All solutions and cleaning procedures were performed with purified water with a Milli-Q system (18.2; Millipore, USA).

### Microelectrophoretic Mobility Measurements

The electrophoretic mobility of erythrocytes or thrombocytes was measured with Zetasizer Nano ZS (Malvern Instruments, UK) apparatus. The measurements were carried out as a function of pH. The cell membranes were suspended in NaCl solution and titrated to the desired pH with HCl or NaOH. The reported values represent the average of at least six measurements performed at a given pH.

From electrophoretic mobility measurements, the surface charge density was determined by the following equation (Alexander and Johnson 1949):

17 $$ \delta = \frac{\eta \cdot u}{d} $$

where η is viscosity of solution, *u* is electrophoretic mobility, and *d* is diffuse layer thickness.

The diffuse layer thickness was determined from the following formula (Barrow 1996):

18 $$ d = \sqrt {\frac{{\varepsilon \cdot \varepsilon_{0} \cdot R \cdot T}}{{2 \cdot F^{2} \cdot I}}} $$

where *R* is the gas constant, *T* is the temperature, *F* is the Faraday number, *I* is the ionic strength of 0.9 % NaCl, and ε and ε_0_ refer to the permeability of the electric medium.

## Results and Discussion

The influence of postmortem changes in surface charge of erythrocyte and thrombocyte as a result of sudden unexpected death was examined. The experimental data of surface charge density were calculated from measured electrophoretic mobility values using Eq. 17, presented in Materials and Methods. The measurements were performed at several pH values, using 0.155 M NaCl as a supporting electrolyte. The theoretical values of surface charge density were determined by applying Eq. 12 to the experimental data.

**Fig. 1:**
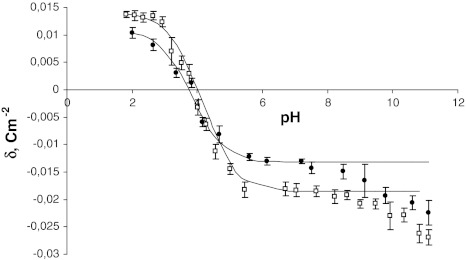
pH dependence of surface charge density of erythrocytes. *Circles* control, *squares* sudden unexpected death. Experimental values are indicated by *points* and the theoretical values by the *curve*

**Fig. 2:**
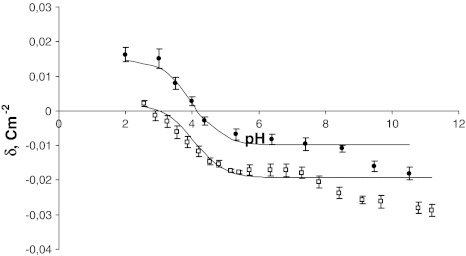
pH dependence of surface charge density of thrombocytes. *Circles* control, *squares* sudden unexpected death. Experimental values are indicated by *points* and the theoretical values by the *curve*

Both experimental and theoretical surface charge density values of the cell membranes as a function of pH are presented in Figs. 1 and 2. The former are indicated by points, and the latter are indicated by curves.

The surface charge densities of the control and the sudden unexpected death erythrocytes are plotted in Fig. 1. If we consider an acid solution, an increased positive charge is observed in erythrocytes after sudden unexpected death in comparison to control erythrocytes. In a basic solution, we also observed an increase in negative charge in erythrocytes after sudden unexpected death in comparison to control erythrocytes and a small shift of the isoelectric point of the membrane to high pH values.

The surface charge densities of the control and the sudden unexpected death thrombocytes are plotted in Fig. 2. In the thrombocytes case, if we consider an acid solution, a decreased positive charge is observed in thrombocytes after sudden unexpected death in comparison to control thrombocytes. In basic solutions, we also observed an increase in the negative charge in thrombocytes after sudden unexpected death in comparison to control thrombocytes and a shift of the isoelectric point of the membrane to a low pH values.

Mathematical calculations based on the four equilibria model (presented in Theory), describing adsorption of electrolyte ions on a cell membrane surface, enabled to quantitative evaluation of the membranes characterizing parameters. The total concentrations of functional acidic ($c_{\text{A}}$) and basic ($c_{\text{B}}$) groups on the erythrocyte as well as thrombocyte surface and their average association constants with hydrogen ($K_{\text{AH}}$) and hydroxyl ($K_{\text{BOH}}$) ions were calculated based on Eqs. 15 and 16, as described previously by Dobrzyńska et al. (2006). The determination of all above parameters was feasible by making an assumption that the $K_{\text{ANa}}$ and $K_{\text{BCl}}$ association constants values are the same as those obtained for phosphatidylcholine liposomes. The $K_{\text{ANa}}$ and $K_{\text{BCl}}$ values of phosphatidylcholine surface groups with sodium and chloride ions were previously reported and are equal to 0.230 and 0.076 m^3^/mol, respectively (Dobrzyńska et al. 2007). The obtained $c_{\text{A}}$, $c_{\text{B}}$, $K_{\text{AH}}$ and $K_{\text{BOH}}$ values were substituted into Eq. 12 to produce a surface charge density versus pH theoretical curves for the studied cell membranes. The parameters characterizing both erythrocyte and thrombocyte surfaces are presented in Tables 1 and 2, respectively. These data were analyzed by standard statistical analyses and are expressed as mean and standard deviation.

**Table 1:** Total concentrations of acidic and basic functional groups of erythrocytes and their association constants with H^+^ and OH^−^ ions

Group	Parameter			
	*c*_A_ (10^−6^ mol/m^2^)	*c*_B_ (10^−6^ mol/m^2^)	*K*_AH_ (10^2^ m^3^/mol)	*K*_BOH_ (10^7^ m^3^/mol)
Control	7.06 ± 0.42	1.54 ± 0.47	3.39 ± 1.12	3.65 ± 0.84
Sudden unexpected death	5.34 ± 0.10	1.68 ± 0.08	6.95 ± 0.73	30.7 ± 0.60

**Table 2:** Total concentrations of acidic and basic functional groups of thrombocytes and their association constants with H^+^ and OH^−^ ions

Group	Parameter			
	*c*_A_ (10^−6^ mol/m^2^)	*c*_B_ (10^−6^ mol/m^2^)	*K*_AH_ (10^2^ m^3^/mol)	*K*_BOH_ (10^7^ m^3^/mol)
Control	3.67 ± 0.79	1.17 ± 0.21	2.81 ± 1.70	2.04 ± 0.59
Sudden unexpected death	6.44 ± 0.08	2.71 ± 0.07	4.67 ± 0.43	25.4 ± 0.56

As can be seen from Figs. 1 and 2, the theoretical and the experimental surface charge density values agree between pH 2 and 9 but diverge in the high pH range. The association equilibria depend on pH that is related to changes of ionic forms of the functional groups involved in the equilibria. Therefore, the observed deviation may be caused by the interactions occurring between the functional groups of the blood cell membrane components, which were not taken into account in the proposed theoretical model. The model describes the equilibria with electrolyte ions only. Biological membranes are complex systems, so it is currently difficult to indicate exactly what interactions may cause the effect of incompatibility of both curves only in the reliable pH range.

The concentration of acidic functional groups in erythrocytes after sudden unexpected death decreased compared with control groups; however, the concentration of basic groups did not change in a statistically significant manner (Table 1). The *K*_AH_ increased two times after sudden unexpected death compared with the control group, whereas the *K*_BOH_ value is eight times higher compared with the control group.

In thrombocytes after sudden unexpected death, we observed an increase in the acidic group’s concentration *c*_A_ and an increase of the basic functional concentration *c*_B_ compared with the control group (Table 2). Sudden unexpected death induces an increase in *K*_AH_ and *K*_BOH_ values in thrombocytes compared with control groups.

Biochemical profiles at autopsy may show considerable case variations due to various factors involving preexisting disorders, cause of death, complications, and environment (Maeda et al. 2009). Luna (2009) postulated that forensic examiners need a real model of cadaveric physiology to understand the differences between living and cadaveric tissue. One of the most important elements of this model is evaluation of membrane changes in blood cells. Our results here demonstrate that the electrical properties of both erythrocyte and thrombocyte membranes are affected by sudden unexpected death. Our experiment demonstrates that alterations in surface charge of the human cell membranes results in variations of all analyzed parameters (*c*_A_, *c*_B_, *K*_AH_, and *K*_BOH_). It is well known that a surface charge is dependent on molecular composition of the cell membrane, particularly of the type and number of surface functional groups. Numerous experiments studying such relationships have been previously performed with on model (Kotyńska et al. 2008; Dobrzyńska et al. 2007) as well as on biological membranes (Dobrzyńska et al. 2008; Szachowicz-Petelska et al. 2008). The analyzed postmortem changes in the surface charge of the membranes compared to control are probably the result of a number of processes occurring in the cells membranes after sudden unexpected death. We suppose that existing interactions between the cell membrane components and between them and their surroundings lead to the appearance of new functional groups on the membrane surfaces and/or to the disappearance of the existing ones, which in turn causes alterations in all analyzed parameters characterizing the cell membrane.

To our knowledge, this is the first report to describe the quantitative changes in cell membrane surface properties after sudden unexpected death. However, our study is preliminary. More in-depth research will provide essential information for understanding biological phenomena.

In conclusion, the interactions between erythrocyte and thrombocyte membranes after sudden unexpected death and solution ions have been characterized. The dependence of the surface charge density of the blood cells on pH was described by using a mathematical model derived from experimental electrophoretic data. The theoretical estimates of electric charge enabled the determination of both total concentrations of acidic and basic functional groups of the analyzed cell membranes and their association constants with electrolyte ions.

We emphasize that there are many problems in diagnosis in forensic medicine—for example, estimation of the time of death. Therefore, we are convinced that knowledge of the equilibria existing within postmortem cell membranes and the processes accompanying them can be helpful in understanding the results obtained by forensic analyses.
